# Climate change and heat stress resilient outdoor workers: findings from systematic literature review

**DOI:** 10.1186/s12889-024-19212-3

**Published:** 2024-06-26

**Authors:** Peymaneh Habibi, Jaleh Razmjouei, Amirhossein Moradi, Farank Mahdavi, Saeed Fallah-Aliabadi, Ahad Heydari

**Affiliations:** 1 Department of Occupational Health Engineering, School of Public Health, Tehran University of Medical Sciences, Tehran, Iran; 2 Health, Safety & Environment (HSE), Shahid Beheshti University of Medical Sciences and Health Services, Tehran, Iran; 3 Safety and Risk Engineering, Faculty of Engineering and Applied Science, Memorial University of Newfoundland, St. John’s, Newfoundland, NL Canada; 4 Department of Health in Emergencies and Disasters, School of Public Health, Shahid Sadoughi University of Medical Sciences, Yazd, Iran; 5 Accident Prevention and Crisis Research Center, Shahid Sadoughi University of Medical Sciences, Yazd, Iran; 6 Department of Health in Disaster and Emergencies, School of Medicine, Kurdistan University of Medical Sciences, Sanandaj, Iran

**Keywords:** Climate change, Resilience, Outdoor workers, Adaptation strategies, Occupational heat stress

## Abstract

**Purpose:**

Global warming has led to an increase in the number and intensity of extreme heat events, posing a significant threat to the health and safety of workers, especially those working outdoors, as they often have limited access to cooling strategies. The present systematic literature review (a) summarizes the current knowledge on the impacts of climate change on outdoor workers, (b) provides historical background on this issue, (c) explores factors that reduce and increase thermal stress resilience, (d) discusses the heat mitigation strategies, and (e) provides an overview of existing policy and legal frameworks on occupational heat exposure among outdoor workers.

**Materials and methods:**

In this systematic review, we searched scientific databases including Scopus (*N* = 855), Web of Science (*N* = 828), and PubMed (*N* = 202). Additionally, we identified relevant studies on climate change and heat-stress control measures through Google Scholar (*N* = 116) using specific search terms. In total, we monitored 2001 articles pertaining to worker populations (men = 2921; women = 627) in various outdoor climate conditions across 14 countries. After full-text assessment, 55 studies were selected for inclusion, and finally, 29 eligible papers were included for data extraction.

**Results:**

Failure to implement effective control strategies for outdoor workers will result in decreased resilience to thermal stress. The findings underscore a lack of awareness regarding certain adaptation strategies and interventions aimed at preventing and enhancing resilience to the impact of climate change on heat stress prevalence among workers in outdoor tropical and subtropical environments. However, attractive alternative solutions from the aspects of economic and ecological sustainability in the overall assessment of heat stress resilience can be referred to acclimatization, shading, optimized clothing properties and planned breaks.

**Conclusion:**

The integration of climate change adaptation strategies into occupational health programs can enhance occupational heat resilience among outdoor workers. Conducting cost-benefit evaluations of health and safety measures for thermal stress adaptation strategies among outdoor workers is crucial for professionals and policymakers in low- and middle-income tropical and subtropical countries. In this respect, complementary measures targeting hydration, work-rest regimes, ventilated garments, self-pacing, and mechanization can be adopted to protect outdoor workers. Risk management strategies, adaptive measures, heat risk awareness, practical interventions, training programs, and protective policies should be implemented in hot-dry and hot-humid climates to boost the tolerance and resilience of outdoor workers.

**Supplementary Information:**

The online version contains supplementary material available at 10.1186/s12889-024-19212-3.

## Introduction

Extreme weather events and severe heat pose significant hazards to the safety and health of workers, leading to increased accidents, mortality, and morbidity during hot climate conditions [1–3]. Global warming presents a new and formidable challenge for most countries [4, 5]. Global climate change substantially affects physiological and perceptual responses through both direct and indirect effects on core body temperature [6], heart rate, skin temperature, and thermal comfort [7–9]. Working in hot and humid environments during long shifts with high physical activity can jeopardize the safety and health of worker populations [7, 10]. Increased exposure to thermal stress among workers in outdoor environments has been documented in tropical and subtropical countries with hot seasons [11]. Exposure to hot working environments, and the resulting elevated physiological and perceptual responses, can lead to occupational heat stress, reducing safety, health, and work capacity [12], and increasing the risk of heat-related illnesses (HRI) [13]. The increment in the levels of ambient temperature, radiation and shifts in the distribution of daily peak temperature can cause indirect and direct effects on outdoor workers [14, 15]. High temperatures and high humidity can exacerbate the effects of physical workload on individuals working outdoors during long shifts in developing and tropical countries [16]. Working in high-temperature and high-humidity environments can have adverse health effects on workers, particularly agricultural workers, construction workers, drivers, sellers, brick-making workers, and daily wage workers [17, 18]. High hot-humid and hot-dry temperatures can lead to occupational heat strain when core body temperature rises above 38 °C [19]. Exposure to heat radiation, either when working outdoors with exposure to the sun or around hot machinery, can greatly increase physiological pressure and lead to reduced work capacity [20].These physiological mechanisms worsen under high climate conditions and climate change, emphasizing the need to identify strategies to increase occupational heat stress resilience and develop solutions and policies to protect the health and safety of outdoor workers [21, 22]. Projected future global warming conditions will dangerously affect the anticipated occupational heat stress resilience of outdoor workers worldwide. There is insufficient knowledge regarding strategies to increase occupational heat stress resilience, necessitating protective measures against heat stress and climate change to reduce health risks and fatalities for future outdoor workers in hot and humid work environments. The findings of this study can inform planning for increasing occupational heat stress resilience, developing heat acclimation strategies, and identifying risk factors to mitigate heat stress caused by global warming, particularly in middle- and low-income communities.

## Materials and methods

### Search strategy

This systematic literature review was conducted following the preferred reporting items for systematic reviews and meta-analyses (PRISMA) guidelines [23]. We searched scientific databases, including PubMed, Scopus, and Web of Science, and identified additional records through Google Scholar. We used Mesh terms in PubMed to identify synonyms for ‘climate change’ and ‘thermal resistance.’ We also consulted specialists to identify relevant keywords. Our search syntax was developed and applied to title, abstract, or keyword queries in selected databases. To ensure the specificity and accuracy of our search strategy, we tested the number needed to read (NNR) in the Web of Science database. We also investigated the references of included studies and searched key journals via Scopus to identify potentially relevant articles. The full search strategy in three main databases has been mentioned in Appendix 1. Our search syntax was as follows:

PubMed: (“heat wave”[tiab] OR “heat stress”[tiab] OR “climate change*”[tiab] OR (climate[tiab] AND change[tiab]) OR “extreme weather”[tiab] OR “extreme heat”[tiab] OR “global warming”[tiab] OR “hot day*”[tiab] OR “warm day*”[tiab]) AND (“heat tolerance“[tiab] OR “heat resilien*“[tiab] OR (heat[tiab] AND resilien*[tiab]) OR (heat[tiab] AND tolera*[tiab]) OR “Heat resistan*”[tiab] OR thermotolerance[tiab] OR “heat endurance”[tiab] OR (heat[tiab] AND endur*[tiab])) AND (worker*[tiab] OR Firefighter*[tiab] OR “fire fighter*”[tiab] OR firem*[tiab] OR “fire m*”[tiab] OR nurs*[tiab] OR operator*[tiab] OR driver*[tiab] OR farmer[tiab]* OR welder*[tiab] OR miner*[tiab] OR employee[tiab] OR laborer*[tiab] OR labour*[tiab]).

### Inclusion criteria

The research question components (PECO) were as follows: P (workers), E (Exposure), C (heat stress), and O (increase occupational heat stress resilience). We included studies that (a) measured physiological and perceptual responses in workplaces and resting environments of workers; (b) studied working populations, including both males and females (healthy and unhealthy populations); (c) assessed the impact of climate change on occupational heat strain, as well as the health, safety, and well-being of workers including work-related variables (income, work type, time), environmental variables (wet-bulb globe temperature (WBGT), relative humidity), physiological variables (heart rate, respiratory, rate of perceived exertion (RPE)), and demographic variables (age, sex, body mass index (kg/m^2^); (d) focused on air temperature, relative humidity (RH), heat waves, solar radiation, climate change, UV radiation, and thermal stress; (e) considered local and international contexts, countries, and workplaces; and (f) investigated workers’ perceptions of climate change, occupational heat strain, and their knowledge and attitudes toward adaptation strategies.

### Exclusion criteria

Studies were excluded if they (a) studied climate change-related phenomena such as storms, cyclones, rainfall, rising sea levels, and drought; (b) evaluated the impact of climate change on plants, crop yields, pest dynamics, soil processes, water availability, and animals; (c) had inaccessible full-texts; or (d) focused on indoor workplaces.

### Screening and selection

We entered all identified studies into EndNote and removed duplicates. One team member (PH) screened studies based on their titles and abstracts, and two members of the research team (AH and PH) independently selected relevant studies by reviewing the full texts. Disagreements regarding study inclusion were resolved through team discussion. We also conducted searches in three key journals: environmental research, urban climate, and global environmental change, but did not identify any additional studies.

### Data extraction and quality assessment

Two team members (AH and PH) independently assessed the eligibility of included studies based on our inclusion and exclusion criteria. They also evaluated the methodological quality of selected studies using the quality assessment tool for studies with diverse designs (QATSDD), which consists of 16 items and is a reliable and valid tool for assessing the methodological quality of various types of studies [24]. Any disagreements regarding study inclusion were resolved through team discussion.

## Results

### Search results

**Fig. 1:**
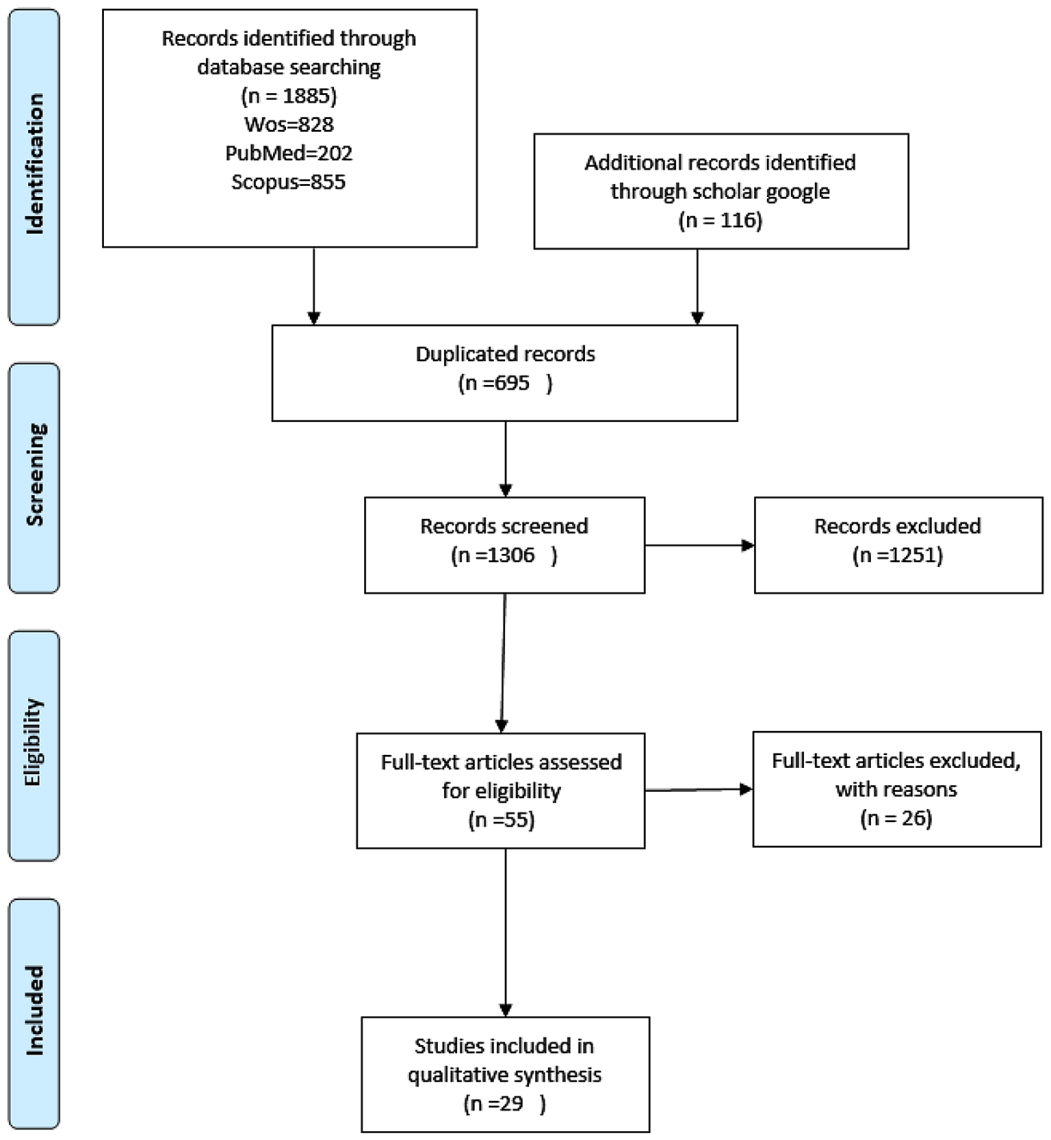
Flow diagram of the screening process of included studies the strategies to increase occupational heat stress resilience among outdoor workers

The numbers of identified studies and the studies reviewed during the screening and selection stages are presented in Fig. 1. The initial search yielded 2001 articles including the additional articles sourced from Scholar Google. After full-text assessment, 55 studies were selected for inclusion, and finally, 29 eligible papers were included for data extraction. No additional studies meeting our eligibility criteria were identified after the full-text investigation. Similarly, no studies were identified through searches of key journals and the references of included studies. Table 1 provides details on the selected studies, including author/year, study location, document type, population/sample size, climate conditions, assessment of physical, perceptual, and physiological factors, authors’ conclusions, and quality ratings. Table 2 presents suggestions for increasing and decreasing occupational heat stress resilience among outdoor workers.

**Table 1:** Characteristics of included studies examining the strategies to increase occupational heat stress resilience among outdoor workers in the context of climate change

NO	Author/year	Study location	Type of document	Population/sample size	Climate conditions	Assessment of physical, perceptual and physiological	Author’s conclusion	Quality rating
1	Bates et al., 2010 [25]	Australia	Cross-sectional	372 M; Manual Workers	Ta = 50 °C	Assessment of hydration status	Ensure that outdoor workers in exposure to hot climate conditions maintain adequate levels of hydration.	32
2	Hanna et al., 2011 [26]	Australia	Review	Different jobs	Hot days (> 35 °C)	WBGT	As global temperatures rise, workers are likely to experience more thermal stress and heat strain, resulting in lower productivity.	34
3	Kim et al., 2011 [27]	USA	Experimental	6 M; Firefighters	Ta = 35 °C, 50% RH	Ta; Tc; HR; Tsk	LCG can be used as an effective method for core body cooling that promotes thermal comfort and heat dissipation during high climate conditions.	30
4	Chan et al., 2012 [28]	China	Cross-sectional	19 M; Rebar workers	WBGT = 32℃	WBGT; Tc; PSI; RPE; HR	Additional rest times, frequency and duration should be introduced between works in high climate conditions to enable workers to recover from exposure to thermal stress.	34
5	Yokota et al., 2012 [29]	USA	Experimental	5 W; Soldiers	Ta = 30 °C, 25% RH Ta = 32 °C, 75% RH	Tc	Fat females tended to have higher core body temperature than medium subjects and lean women maintained lower core body temperature than female soldiers.	34
6	Chan et al., 2013 [30]	China	Review	Construction workers	N	RPE; WBGT	Alcohol drinking, work duration, and age are important predictors to determine the physiological responses among construction workers.	32
7	Yamazaki et al., 2013 [31]	Japan	Review	Different jobs	N	Heat acclimatization	For different jobs that work under high thermal stress, better management of the work rate, heat acclimation, and health is required.	34
8	Kjellstrom et al., 2014 [20]	South Africa	Review	Different jobs	N	WBGT	The climate change model indicates that the thermal stress exposure may increase (2–4 °C) during the hot climate conditions, and this would change the occupational heat stress to high risk.	30
9	Lui et al., 2014 [32]	USA	Experimental	26 M; Firefighters	Ta = 43.3 °C, 33% RH	Tc; Tsk; HR; PSI; RPE	The long-term thermal stress exposure accrued by the wildland firefighters was adequate to obtain heat acclimatization.	28
10	Hatvani et al., 2016 [33]	Australia	Experimental	29 Outdoor workers	N	N	Workers with pre-existing health conditions were the most vulnerable and those were not aware of their vulnerability.	30
11	Park et al., 2017 [34]	Korea	Descriptive study	47 Outdoor workers; 43 M and 4 W	N	WBGT	Heat acclimation during work is the major determinant of HRI.	23
12	Tjaša et al., 2017 [35]	Slovenia	Descriptive study	86 agricultural advisers and 230 farmers	N	N	Climate change impacts on outdoor workers revealed that perceived the thermal stress as discomfort and unsuitable, experienced HRI.	28
13	Yang et al., 2017 [36]	Taiwan	Cross-sectional	12 M; Coal-fueled power plant and heavy-oil power	Ta = 30–40 °C	WBGT Tc; Tsk; HR; SWreq	Climate change can be negatively impacted outdoor workers’ safety and health in subtropical countries.	33
14	Kemala et al., 2018 [37]	Indonesia	Cross-sectional	100 M; Construction workers	Ta = 27 °C	WBGT; PSI;	There was a significant relationship between occupational heat strain with water intake adequacy rate, acclimatization, and BMI.	22
15	Nunfam et al., 2018 [38]	Ghana	Review	Working population	N	WBGT; Questionnaires	Adequate studied and research should be conducted to develop policies to combat the threat of high thermal stress to enhance workers’ resilience to climate change and increase adaptive capacity.	32
16	Nunfam et al., 2019 [39]	Ghana	Cross-sectional	15 M and 1 W; Mining workers	N	Questionnaires	An effective workplace thermal management policy requires an adequate understanding of occupational heat strain and stress risks, continued training, adaptation strategies, and education among mining workers.	28
17	Nunfam et al., 2019 [40]	Ghana	Review	Different jobs	N	N	Outdoor workers can reduce thermal stress, boost resilience, and efforts to achieve sustainable development goals.	29
18	Nunfam et al., 2019 [16]	Ghana	Cross-sectional	Different jobs	N	Questionnaires	Occupational heat stress prevention strategies should focus on promoting workers’ adaptation strategies and inform policy decisions.	32
19	Kim et al., 2020 [12]	Korea	Cross-sectional	Different jobs	Ta = 27.85 ℃ WBGT = 26.66 ℃	WBGT	The need for providing an adaptation policy and considering the occupational structure related to work capacity in tropical countries.	26
20	Nunfam et al., 2020 [41]	Ghana	Cross-sectional	144 M and 17 W; Mining workers	N	N	Adaptation policy and social protection strategies should be considered to reduce workers’ vulnerability to thermal stress.	29
21	Talebi et al., 2020 [42]	USA	Cross-sectional	Underground Mine Workers	N	N	Acclimated workers with clothing can have more continuous work (a full 8-h shift) compared with non-acclimated workers (less than 5 h).	23
22	Tigchelaar et al., 2020 [43]	USA	Cross-sectional	Agricultural workers	N	N	Safeguarding the well-being and health of agricultural workers require systemic change such as workplace adaptations for the mitigation of heat strain.	26
23	Han et al., 2021 [44]	China	Cross-sectional	239 M and 79 W Construction workers	N	Questionnaire	There is a need to improve the awareness of workers about HRI and strengthen high temperature related to the current heat prevention policies, training, and education.	32
24	Pal et al., 2021 (Pal, Patel, & Banik, 2021)	India	Review	Agricultural Workers	N	N	Develop better strategies and adaptation policies preventing the effect of thermal stress due to climate change on agricultural workers.	32
25	Tang et al., 2021 [45]	China	Cross-sectional	1063 M Construction Workers	Mean (maximum) Ta = 38.84 °C (47.19 °C), 67.7% (93.8%) RH	Questionnaire	Outdoor workers need to be modified according to the hot climate conditions to avoid excessive exposure to warm and high-temperature work environments.	29
26	Venugopal et al., 2021 [46]	India	Cross-sectional	532 M and 521 W; Outdoor workers	WBGT = 37.5 ℃- 49 ℃	WBGT	Protective policies, adaptive strategies, and proactive mitigation efforts are needed to avert health for workers in developing nations.	32
27	Habibi et al., 2021 [47]	Iran	Systematic review	Different jobs	N	N	Strong evidence shows that managing the adverse effects of thermal stress on outdoor workers should be considered.	32
28	Butt et al., 2022 [48]	Pakistan	Review	Different jobs	N	N	Adaptation policies are required to acclimatize the workers in hot climate conditions.	23
29	Hunt et al., 2023 [13]	Australia	Cross-sectional	Different jobs	N	WBGT	Risk management strategies must adapt to hot climate conditions to protect outdoor workers from the effects of thermal stress.	32

WBGT = Wet Bulb Globe Temperature; Tc = Core Temperature; HR = Heart Rate; LCG = Liquid Cooling Garments; Tsk = skin temperature; Ta = Dry Temperature; PSI = Physiological Strain Index; RPE = Ratings of perceived exertion; RH = Relative Humidity; HRI = heat-related illness; SWreq = Required Sweat Rate; N = Not Mentioned

**Table 2:** Characteristics of included studies examining the strategies to increase and decrease occupational heat stress resilience among outdoor workers in the context of climate change

Author/year	Factors that reduce thermal stress resilience	Factors that enhance thermal stress resilience
Bates et al., 2010 [25]	Dehydration.	Programmes to improve hydration status.
Hannaet al., 2011 [26]	Work intensities; Clothing types; Dehydration; Unacclimatized; Physically unfit; Poor ventilation; High humidity; Slow air movement over the skin; PPE; Less fit; Air pollution (especially ozone); Heat radiation; Renal failure; Cardiac failure; Caffeine and alcohol; Air temperatures; Increase in core body temperature; Age.	Work-to-rest ratios; Aerobic fitness; Body fatness; Drinking sufficient water and electrolytes; Acclimatization; Hot spots; Educate employers.
Kim et al., 2011 [27]	SCBA; Hot and humid environments; Increase in core body temperature; NBC; Physical activity.	Cooling vests with ice packs; Phase changing materials; Cooling fans; Cold water immersion; LCG.
Chan et al., 2012 [28]	Smokes; caffeine; alcohol; Elderly workers; Poor ventilation; Dehydration.	Recovery time; Shade/ shelter and cooling device; Sufficient cool (10–15℃) drinking water at easily accessible drinking points; Avoid working under direct sunlight; Wear light-colored; Loose fitting; Long-sleeved clothing; Ventilated helmets; Educate employers; Work-rest schedules.
Yokota et al., 2012 [29]	Body fat; BMI; Body surface area; Unacclimated; High core temperature.	Acclimatization; Normal anthropometric.
Chan et al., 2013 [30]	Smoking and alcohol-drinking habits; Age; Resting heart rate; Percentage body fat; Work duration; Air-pollution; Energy consumption; Respiratory exchange rate; Air-pollution index.	Acclimatization; TWL.
Yamazaki et al., 2013 [31]	Protective clothing; Increase in core body temperature; Environmental conditions (temperature, humidity, radiant heat, etc.); Physical work (rate and duration of work); Age.	Exercise training; Acclimatization; Fluid intake.
Kjellstrom et al., 2014 [20]	Chronic kidney disease; Dehydration.	Easy access to safe drinking water; Access to medical attention; Training of workers.
Lui et al., 2014 [32]	Unacclimatized; Age; Less fit; Work in a hot environment for a long hour; HRI; Increase in core body temperature; Physical activity; irregularities in hydration or electrolyte status; Body fat.	Acclimatization.
Hatvani et al., 2016 [33]	Age; Gender; People with pre-existing health conditions; People with poor health.	Drink plenty of water; Keep shadings; Acclimatization.
Park et al., 2017 [34]	Environmental factors; Metabolic heat; Clothing; Heat injury; High air temperature; Minimal movement of air; High humidity; Radiant heat; Physical work; Heavy work; Dehydration; Time of exposure to heat stress; Medical characteristics; Health status; High dry bulb air temperature; Age; Experience of a previous HRI; Sleep disorders; Severe obesity; Increased core temperature and pulse Rate; Consumption of alcohol.	Wearing a conventional one-layer work clothing (long-sleeved work shirt and trousers); Acclimatization.
Tjaša et al., 2017 [35]	Total working hours; Age; Gender; Chronical disease; PPE.	Educational programs; Regular breaks; Drinking water; Working in the shade; Wearing light colored and permeable clothes; Taking breaks in the shade; Responding to early symptoms; Taking a break in a cooler space; Changing to lighter/less clothing; Wearing broad-brimmed hats; Reschedule working hours.
Yang et al., 2017 [36]	High radiant heat.	Work-rest schedules; Experienced and acclimatized workers.
Kemala et al., 2018 [37]	BMI; Age; Work period; Health Status; Alcohol / Drugs Consumption; Chronic hypo hydration; Less cardiac function.	Acclimatization; Water intake adequacy rate.
Nunfam et al., 2018 [38]	Pregnancy; Lack of training about thermal stress risks; Gender; kidney disease; High heat exposures; Heavy workload; Older workers with low education; PPE.	Improve guidelines and occupational health standards; Regular breaks in shaded areas; Training of workers on HRI prevention; Understand ability to self-pace; Improve ventilation and install air cooling devices; Wear sun protective gear; wear a wide-brimmed hat; use of fan and sunblock; provision of a central cooling system; electric fans use.
Nunfam et al., 2019 [39]	Gender; Age; Education; The amount of air moisture in outdoor setting/workplace; Duration of working hours; Heat radiation from the sun and other sources around the workplace; Type of physical workload; Type of protective clothing; HRI concerns; Excessive sweating.	Awareness of climate change; Access to cooling systems (e.g., air conditions & fans); Duration of break/rest hours; Access to drinking water; Access to shade; Wearing loose and light-coloured clothing.
Nunfam et al., 2019 [40]	Pregnancy; Lack training; Older workers; Low education; Gender; Temperature; Air movement; Humidity; Solar radiation; Physical workload; Clothing; Type of work; Age; Body size; Medical condition; Medication; Use of drugs and alcohol; Physical Fitness; Metabolism rate, Choice of clothing; Prior heat injury.	Heat risk awareness; Heat education and training; Self-pace; Work-break regimes; Shade; Cooling systems; Acclimatization; Rehydration.
Nunfam et al., 2019 [16]	Age; Level of education; Workload; Years of working experience; Job physically demanding; Frequency of work around heat sources; Increase in temperature and hot environment; The amount of air moisture in the outdoor settings or workplaces; Airspeed/movement around the workplace; Heat radiation from the sun and other sources around the workplace; Type of physical workload; The duration of working hours; Type of protective clothing; Gender.	Awareness of climate change; Access to the cooling system, e.g., air conditioning and fans; Duration of break/rest hours; Access to shade; Access to drinking water.
Kim et al., 2020 [12]	High temperature.	Adaptation policies governing heat exposure; Employment standards and regulations; Adjustment of scheduled breaks.
Nunfam et al., 2020 [41]	Gender; Age; Level of education; Years of working experience; Workload; Working hours; Workplace environment; Work around heat sources; Frequency of work around heat sources; Drink coffee, soft drinks, caffeinated energy drinks and alcohol; Inadequate knowledge of coping and adaptive behaviour; Lack of regular training on heat stress risk assessment, work safety and adaptation measures; Lack of specific heat-related policies and regulation; Poor compliance and implementation of heat stress guidelines, policies and programme; Inadequate financial resources to support engineering control; Lack of management commitment; Lack of access to innovative technology and equipment.	Frequently drink lots of cool water before feeling thirsty; Wear loose and light-coloured clothing; Take regular breaks away from hot conditions in a cooler or shaded area; Used to working in the heat without any medication to cope with heat stress; Use mechanical equipment; Plan and carry out heavy routine outdoor work during the early morning or evening hours or in shaded areas; Training programmes; Share unavoidable heavier jobs and rotate jobs; Slow down work at my pace; Use PPE like sunglasses, wide-brimmed hats and hand gloves; Use cooling systems; Adaptation policy.
Talebi et al., 2020 [42]	Clothing insulation; Age; Gender; Fitness; Lifestyle; Experiences; Past medical history; Air temperature; Air velocity; RH; Radiant temperature; Metabolic rate; PPE; Dehydration level; Elevated heart rates and Core temperatures.	Acclimation.
Tigchelaar et al., 2020 [43]	PPE; UV-radiation; Absent appropriate training and advances; Age; Dehydration; Kidney injury; Chronic diseases; The absence of shade; Limited opportunities to adequately hydrate; Minimizing breaks; Air temperature; Humidity; Solar radiation; Wind speed.	Reduction of carbon emissions; Acclimation; Increases in rest time; Changing clothing ensembles to a more breathable single-layer garment; Work/rest cycle; Taking breaks in an air-conditioned environment.
Han et al., 2021 [44]	Gender; Age; Education level; Workplace environment; Physically demanding; Work close to heat sources; PPE (e.g., reflective vests, safety boots, and gloves); Heat illness experience; Heat-related injury experience; Culture/religion.	Drinking water; Training; Rescheduling working hours (e.g., starting work early, extending rest time); Stopping working when the temperature exceeded 40 °C; Air conditioning or central cooling system; Shaded rest area; Electric fan; Sunscreen cap; Acclimatization; Self-pacing; Wearing light-colored breathable clothes.
Pal et al., 2021 (Pal, Patel, & Banik, 2021)	U.V. radiation; Heatwaves; HRI; Hot environment; High humidity; Physical exercise; Dehydration; Clothing; Relative air velocity; Mean radiant temperature; Air temperature; Increase in core body temperature; Increase in skin temperature; Increase in blood pressure; body mass loss; Increase in heart rate; Chronic kidney; Cardiovascular illness; Lack of proper health education; Increase in oral temperature.	Medical training; Adaptation strategies; The provision of proper shade for resting; Availability of drinking water at the workplace; Conducting training and awareness program; Use of cooling mechanisms; Shifting of work; Use of supportable protective equipment.
Tang et al., 2021 [45]	Increased heart rate and body temperature; High blood pressure; Skin cancer; Allergic diseases; Temperature; Mean radiation temperature; Humidity; Air velocity; Solar radiation; Metabolic rate.	The workload should be reduced when the outdoor temperature exceeded 34 °C.
Venugopal et al., 2021 [46]	HRI; High ambient temperatures; Heavy workload; Long years of heat exposures; Reduced kidney function; Age; Gender; Smoking status; Alcoholic status; Duration of employment; Dehydration; Change in urine volume/color; Alcohol; Education; Intense physical activity; Cardiovascular diseases; Mental health problems; Chronic Kidney Disease; PPE; The lack of awareness about the risks of heat stress; Solar radiation; Core temperature elevation; lack of workplace regulations (duration of work, and improper work/rest schedule and appropriate welfare facilities).	Adaptive strategies; Training; Protective workplace policies; Cooling interventions.
Habibi et al., 2021 [47]	Age; BMI; Gender; Aerobic capacity; PPE; Education level; Obesity; Fitness level; Medical conditions; Heart disease; Lung disease; Pregnancy; Respiratory disease; Diabetes; Cardiovascular disease; Chronic disease; Health status; Kidney disease; Infection disease; Disabilities; Sensitivity of individuals; Dehydration or poorly hydrated; Skin temperature; Core body temperature; Sustained sweating; Insufficient in sweat rate; Heat illness history or injury; Hypertension; Reducing the body’s ability to cool itself; Skin problem; Liver problem; Reproductive hormones; Menstrual phase; Breast-feeding; Malnutrition; Thyroid disease; Immunologic status; Mental illness; Cognitive impairment; Psychological distress; Psychiatric illnesses; Degree of acclimatization; Metabolic rate; Insufficient fluid replacement; Drugs and alcohol exposure; Smoking; Nephrotoxic drugs; Excess use of NSAIDs; Caffeine consumption habit; Use of medical drugs; Social stress; Ergonomic risks (Body posture, Movement, Confined space and etc.); Workplace pressure; Environmental conditions (Air temperature, Heatwave, Extreme weather and etc.); Tropical and subtropical countries; Atmospheric pressure; Solar radiation; U.V. radiation; Heat radiation; Relative humidity; Air pollution; Air movement; Waste generation; Dew point temperature; Income; Individual work habits; Work characteristics (Task complexity, Concentration and etc.); Poor working conditions; Physical demands of jobs; Working around heat sources; Exposures to pesticides; Exposure to toxic and highly evaporative chemicals; Expanded vector habitats; Clothing properties(Protective clothing, Color of clothing and Size of clothing); Low-price clothes, shoes, furniture, and other consumer products; Poorly or no air-conditioning; Poor access to safe drinking water; Absence of trees and vegetation in urban areas; Exceed in ACGIH TLV, WBGT; Work shift; Time spent indoors/outdoors; Duration of break/rest hours; Inadequate prevention and control policies; Middle and low income countries; Inadequate awareness of heat stress risks, training and skills; Lack of occupational health and safety programs.	Education and training; Use and improving of guidelines, risk assessment, indices and standards; Use of preventive strategies in warm climate; Work/rest cycle; Adequate supply of clean drinking water; “Stopping work” in exposure to hot temperature(> 40 ◦C); Responding to early symptoms; Policy and regulation implementation; Selection criteria when recruiting workers; Temporary tents for rest; Global ‘Hothaps’ programme; Rearrangement of work tasks to cooler parts of the day and season; Proper mechanical aids; Identify injuries, illnesses, and deaths on hot days; Use of appropriate PPE; Proper a cold rest places; Proper personal water bottles; Job rotation; Reducing metabolic rate; Keeping trees or other creators of shade(roof or walls); Apply for a max threshold to heat exposure in countries; Avoiding direct sunshine; Increase in sweating; Adjust work activities; Proper air movement around the skin; Medical monitoring; Provides motivation for employers; Nutritional status; Widespread precautious work; Cooling, air conditioning and electric fans; Reduce in greenhouse gas emission; Heat-Shield project; Early warning and emergency response systems; Eliminating or reduction heat sources; Design and insulation of workplace buildings; Reducing humidity; Increasing air velocity; Application of occupational health principles; Engineering and administrative controls; Personal cooling techniques; Adjust working schedule; Wearing large hats or cap; Self-pacing; Acclimatization; Open windows or use of natural cooling systems; Bathing in cold water; ‘Buddy system’.
Butt et al., 2022 [48]	Air temperature; Polluted air; U.V. radiation; Increase physiological strain; Hyperthermia; High dynamic physical work; Excess water loss; Elderly workers; Heat exhaustion; Dehydration; Poor ventilation and cooling system; Relative humidity; Air velocity; Solar radiation.	Protective policies and actions; Adaptation policies.
Hunt et al., 2023 [13]	Air temperature; Higher air humidity; Limits body heat dissipation; Protective clothing; Physical effort; Elevated metabolic heat production; HRI; Heavy work; Increase in deep body temperature; Dehydration; Sweat loss; Low wind speed; BMI; Continuous work without a break permitted; Elevated heart rate; Cardiovascular strain; heat waves; Fitness; Age.	Risk management strategies; Work duration limits based on core temperature elevation; Fluid ingestion; Drinking freely; Heat acclimatization; Monitoring daily environmental conditions; Work–rest scheduling; Adjusting work intensity; Cooling strategies; Self-pace tasks; Rescheduling work to cooler parts of the day; Allowing longer rest periods; Resting in the shade; Wearing short-sleeved shirts and shorts may be reduced.

SCBA = Self-Contained Breathing Apparatus; NBC = Nuclear, Biological, and Chemical clothing; BMI = Body Mass Index; TWL = Thermal Work Limit index; HRI = heat-related illness; RALs = Recommended Alert Limits; RELs = Recommended Exposure Limits; TLV = Threshold Limit Value; PPE = Personal Protective Equipment; U.V. radiation = Ultraviolet radiation; NSAIDs = Nonsteroidal Anti-Inflammatory drugs

### Descriptive analysis

Out of the 29 selected studies, 18 addressed global warming’s impact on occupational heat stress resilience, risk management strategies, and adaptation strategies for warming conditions. Most of these studies emphasized that climate change will exacerbate the health impacts of extreme heat. The prevalence of negative effects due to climate change will intensify workers’ health risks in future work scenarios, particularly in regions with hot and humid climates and poor economic conditions. As of our selection period until 2023, 20 studies (68.96%) were published between 2016 and 2023. Of the 29 assessed papers, 18 (62.06%) directly investigated the effects of climate change and adaptation strategies for outdoor workers in various countries, including Australia, the USA, China, Japan, Africa, Korea, Slovenia, Taiwan, Indonesia, Ghana, Korea, India, Iran, and Pakistan. The predominant themes identified in these papers revolved around strategies to increase occupational heat stress resilience. In conclusion, the study’s findings were categorized into main themes, including risk factors that decrease occupational heat stress resilience and suggestions for increasing occupational heat stress resilience among outdoor workers.

### Thematic content analysis

This systematic review provides a summary of evidence published to date regarding strategies to enhance occupational heat stress resilience, especially in hot outdoor workplaces. Despite variations in study design and analytical approaches, the evidence presented in this systematic review consistently highlights a strong association between thermal stress resulting from global warming and occupational heat stress. Broad findings from these studies indicate that exposure to heatwaves and global warming is linked to adverse health impacts on workers.

Furthermore, several studies underscore the need for sentinel effects and leading indicators to facilitate surveillance of climate-related occupational heat stress effects, as well as strategies and interventions for preventing the impact of climate change on outdoor workers. Finally, the review identifies interventions and adaptation strategies for outdoor workers, including the provision of accessible cool drinking water [13, 26, 41, 44, 47], optimized work-rest schedules [12, 13, 16, 26, 36, 43, 44, 47], the availability of proper resting shade [16, 47, 49], training and awareness programs [20, 38, 40], self-paced work [13, 38, 40, 44, 47], and the use of supportive protective equipment [41].

### Factors that reduce resilience to climate change among outdoor workers

Resilience to climate change among outdoor workers can be reduced by various factors, categorized into personal risk factors, environmental risk factors, and occupational-related heat exposure risk factors during work.

#### Individual-related heat exposure risk factors

Personal factors associated with reduced resilience to climate change, identifiable from outdoor workers’ data, include dehydration [20, 25, 28, 32, 34, 37, 40, 46–48], unique medical characteristics [41, 47], pregnancy [38, 40, 47], BMI [29, 30, 37, 40, 47, 49], obesity and body fat [29, 30, 32, 34, 47], overall health status [33, 34, 37, 47], lack of sleep [33, 34, 40, 47], experience of a previous HRI [32, 34, 44–47], presence of certain concurrent diseases and chronic disease [35, 47], kidney disease [20, 26, 38, 43, 46, 47], consumption of caffeine and alcohol [26, 28, 30, 34, 37, 40, 41, 46, 47], smoking [30], use of drugs [26, 37, 40, 41, 47], age [16, 33, 35, 38–41, 46, 47], older workers with low education [38, 40, 43, 44, 46, 47], physical fitness [26, 32, 40, 47], metabolism rate [40, 47], type of clothing [40, 47], prior heat injury [40, 46, 47], physical activity and heavy workload [16, 27, 31, 34, 38–40, 46–48], gender [16, 33, 35, 38–41, 46, 47], education level [16, 39, 41, 44, 46, 47], wearing PPE [16, 26, 27, 31, 38, 39, 44, 46, 47], and non-acclimatization [29, 32, 37, 40, 41, 43, 44, 47]. Physiological risk factors most frequently expressed by outdoor workers included excessive heart rate [30, 45, 47, 49], oral [47, 49], skin [45–47, 49], core temperature [26, 27, 29, 31, 32, 34, 45–49], sweating [39, 47], and blood pressure [45–47, 49]. This is often followed by heat exhaustion [47, 48] or tiredness [47], headaches [47], heat rash [47], and fainting [47]. Older adults are more vulnerable to chronic dehydration [28, 45], especially those living with multiple chronic diseases [43, 47]. Aging is also associated with reductions in sweat production [8]. Consequently, studies have generally reported greater elevations in body heat storage and core temperature in older compared to younger adults during environmental heat exposure [26, 37, 46, 47]. Additionally, personal factors correlated with occupational heat strain include the adequacy of water intake [41, 47].

#### Environmental-related heat exposure risk factors

The environmental factors contributing to thermal stress include high air temperature [12, 16, 26, 27, 31, 34, 40, 45–49], heat wave [43, 47, 48], airspeed and movement around the workplace [16, 43, 45, 47–49], high levels of heat exposure (WBGT = 37.5–49 ℃) [38, 46, 47], tropical nights [48], working in sun- exposed conditions [16, 38, 39, 47], solar radiation [26, 40, 43, 46–48], high humidity [16, 27, 31, 34, 40, 43, 45, 47–49], UV radiation [26, 47–49], the moisture content of the outdoor settings or workplaces [16, 39], radiant heat [16, 26, 31, 36, 45, 47, 48], and the air-pollution index [30].

#### Occupational-related heat exposure risk factors

However, workers encounter various barriers, such as inadequate cool housing designs for rest [38], a lack of management and engineering commitment [41, 42, 47], heavy physical workloads for long hours [16, 47] or physically demanding jobs [44, 46], insufficient awareness and prevention training [38, 40, 41, 43, 47], a lack of knowledge regarding adaptive behavior [41, 43], the absence of occupational heat stress guidelines and adaptation strategies [38, 41, 46, 47], a lack of regular training on adaptation measures [41], limited management commitment [41], the nature of the physical workload [16, 40, 41, 46, 47], the absence of specific thermal stress-related policy regulations [41], working in proximity to heat sources [16, 44, 47], the type of protective clothing [16, 40, 47], limited access to innovative technology and equipment [41], the nature of the work [40, 41, 46, 47], inadequate management commitment, work-break regimes [43, 47] and cooling systems [26, 28, 40, 41, 47, 48]. Additionally, workers face challenges such as inadequate knowledge of adaptive behavior [41, 46], a lack of regular training on thermal stress risk, adaptation, and safety measures [41, 47], a deficiency in specific heat-related policies and regulations [41], limited management commitment to heat-related health and safety measures [41], restricted access to innovative equipment and technology [41], insufficient regular breaks and work-rest time [35, 39, 41, 46, 47], limited access to shade [38, 43, 47], inadequate financial resources [38, 41], the absence of an acclimatization program [41, 43, 47], suboptimal water management [47], and insufficient medical attention when implementing adaptation strategies for climate change and occupational heat stress.

### Factors that enhance resilience to climate change among outdoor workers

Enhanced resilience to climate change can be achieved through various means, including personal, managerial, and engineering protective factors.

#### Personal protective factors

Outdoor workers can take several measures to protect themselves. They should consider adjusting their work schedule [35, 47], maintaining adequate hydration [28, 33, 35, 37–40, 47], adjusting their clothing [31, 35, 47], drinking more water [35] or drinking plenty of cool water frequently before feeling thirsty [13, 26, 41, 44, 47]. It’s important to take more frequent planned breaks [35, 38, 44], wear broad-brimmed hats [35, 39, 41, 47] and ventilated helmets [28], understand how to self-pace [13, 38, 40, 44, 47], wear sun-protective gear [38, 49], including sunglasses and gloves during hot weather conditions [41], and take work breaks and rest in cooler or shaded areas [13, 28, 33, 35, 38–41, 43, 44, 47]. Using sunblock [38, 39, 44], and having a higher education level [39, 44, 47], are also beneficial. Workers should consider wearing loose and light-colored clothing [28, 34, 35, 38, 39, 41, 44] and opting for short-sleeved shirts and shorts when possible [13]. Using cooling vests [27, 47], implementing a ‘Buddy system’ [47], acclimatization [26, 29–34, 36, 37, 40, 47], maintaining normal anthropometric measures [29], and changing clothing ensembles to more breathable single-layer garments [43] can further enhance personal protection.

#### Managerial protective factors

Maintaining good quality working conditions and a suitable climate can significantly improve worker performance, productivity, and company profits [37]. Workplace management and training programs [16, 35, 38, 40, 41, 47, 49] are crucial for worker well-being. Developing prevention strategies [12, 13, 38, 48], improving guidelines for worker safety, health, and productivity, and adhering to occupational health standards [12, 38, 47] are essential. Scheduling heavy routine outdoor work during the early morning [47] or evening hours or in shaded areas [13, 41, 44, 49] can help mitigate heat stress. Providing access to cooling systems, such as air conditioning and fans [13, 16, 26, 38–41, 44, 47], and offering climate change adaptation strategies [13, 48, 49] are beneficial. Adjusting the duration of breaks/rest periods [12, 13, 16, 26, 36, 43, 44, 47], ensuring access to shade [16, 47, 49], and providing access to drinking water or implementing programs to improve hydration status [13, 16, 20, 25, 44, 47, 49] are important managerial measures. Training workers in heat-related illness prevention [20, 38, 40], providing access to medical attention [20], sharing heavier jobs and rotating job assignments on shift schedules [13, 41, 47, 49], offering air-conditioned vehicles [13, 38, 47], promoting climate change awareness to support healthy lives and decent jobs [39], implementing work stoppages if the daily maximum temperature exceeds 40 °C [13, 44, 47], raising worker awareness about heat risks [47] modifying work habits [49], considering the TWL [30], and promoting the understanding of the need for workers to self-pace during hot weather [13, 38, 47] are all valuable managerial protective factors.

#### Engineering protective factors

Providing and designing regular breaks in shaded areas [38, 47], implementing strategies to eliminate or replace thermal stress risks [37, 44], installing a central cooling system [13, 44, 47], halting work during periods of high thermal stress and supplying mechanical equipment [41, 47], initiating heat-shield projects [47], and enhancing ventilation [38, 39, 44, 47].

## Discussion

Our systematic review’s outcomes help us understand strategies for increasing occupational heat stress resilience and assessing the effects of global warming on outdoor workers’ adaptation strategies. This is particularly crucial in numerous warm workplaces, especially in low- and middle-income countries. The implementation of strategies to ensure adequate hydration, including access to drinking water and programs to improve hydration status [13, 16, 20, 25, 44, 47, 49], is one of the most critical interventions for managing warm workplaces. Hydrated workers [28, 33, 35, 37–40, 47] are more likely to maintain an acceptable work rate and physical activity without health risks in various hot-dry and hot-humid weather conditions [25]. Employers bear the responsibility of providing a safe work environment, conducting training and awareness programs [16, 35, 38, 40, 41, 47, 49], supervision [50], and providing suitable protective equipment to mitigate the negative effects of thermal stress due to global warming on safety and health [26, 49]. Cooling the core body temperature through wearable liquid cooling garments (SCG) [27], evaporative cooling garments (ECGs) [15], fluid cooling garments (FCGs) [51], hybrid cooling (HBCGs) [52], and phase change materials (PCMs) [53] worn by individuals who require personal protective equipment [47, 54], including firefighters and construction workers, significantly reduces occupational heat strain and enhances thermal comfort and performance [32]. Chan et al. recommend implementing appropriate protective measures, such as work-rest schedules and heat tolerance guidelines, to ensure the safety and health of personnel exposed to hot weather conditions [28]. Therefore, it’s advisable to conduct further research on work-rest schedule optimization models for workers, particularly in the context of construction workers [28]. It is recommended that safe work durations should be modified based on expected type of clothing and work intensity [55]. Our review’s results indicate that personal risk factors such as dehydration [20, 25, 28, 32, 34, 37, 40, 46–48], smoking [30] and alcohol-drinking habits [26, 28, 30, 34, 37, 40, 41, 46, 47], age [16, 33, 35, 38–41, 46, 47], BMI [29, 30, 37, 40, 47, 49], and non-acclimatization [29, 32, 37, 40, 41, 43, 44, 47]; as well as work-related factors like work-rest cycles [35, 39, 41, 46, 47] and environmental risk factors such as air temperature [12, 16, 26, 27, 31, 34, 40, 45–49], relative humidity (RH) [16, 27, 31, 34, 40, 43, 45, 47–49], heat radiant [16, 26, 31, 36, 45, 47, 48], and Thermal Work Limit (TWL) [30], are significant predictors for determining the physiological responses to HRI among outdoor workers [30]. More efforts should be made to educate workers and employers about the effects of occupational heat stress on safety, health and performance, and appropriate screening protocols (pre-employment and periodic examinations) should be included in health and safety legislation [56].

Educating outdoor workers about physiological and perceptual responses to HRI [20, 38, 40] and heat acclimation under uncompensated thermal stress [26, 29–34, 36, 37, 40, 47], as well as emphasizing cooling techniques and fluid intake [28, 33, 35, 37–40, 47], is essential. Furthermore, it’s necessary to investigate the impact of gender (both women and men) [16, 33, 35, 38–41, 46, 47] and aging on heat tolerance and psychophysiological adaptation during work in hot-dry and hot-humid environmental conditions. This is especially crucial since elderly workers [38, 40, 43, 44, 46, 47] display increased susceptibility to HRI in future studies, even if they haven’t engaged in prolonged or strenuous physical labor [31]. Pogačar et al.‘s study revealed that the most common symptoms of heat stress include excessive sweating, thirst, and fatigue. Interestingly, there was a significant difference among age groups regarding thirst and excessive sweating [35]. Gender differences in temperature regulation become more apparent under varying heat loads [8]. In general, women lose more heat through convection [11], which is advantageous in hot-humid environments [57], while men lose more heat through evaporation, which is more pronounced in hot-dry environments [58]. The resilience of vulnerable worker groups to heat stress can be compromised despite existing standards and knowledge. This vulnerability is particularly relevant when considering outdoor workers exposed to different climate conditions in tropical and subtropical countries [12, 38, 47]. Kjellstrom et al.‘s study underscores that mine workers remain the most significant population in terms of preventing the impact of thermal stress. This also extends to many construction workers, agricultural workers, and individuals laboring in warm workplaces without effective cooling systems [20]. Lui et al. demonstrated that wildland firefighters experience heat acclimatization across the thermal stress and fire season, leading to significant decreases in physiological and perceptual responses. These adaptations can reduce the risk of HRI [32]. Implementing acclimatization [26, 29–34, 36, 37, 40, 47] and adaptation programms [13, 48, 49] for workers exposed to thermal stress is crucial. Adaptation policies aim to increase climate change resilience and reduce climate vulnerability [48]. Managers and occupational health professionals should also assess workers’ health status and individual habits, such as sleep deprivation [33, 34, 40, 47], dehydration, and alcohol consumption before work [34]. International agencies have proposed various climate change adaptation and prevention strategies, including conducting training and awareness programs, using cooling mechanisms [13, 16, 26, 38–41, 44, 47], and ensuring the availability of cool drinking water [13, 16, 20, 25, 44, 47, 49]. The most effective solutions at mitigating occupational heat strain were heat acclimation [26, 29–34, 36, 37, 40, 47], wearing specialized cooling garments [27, 47], cold water immersion [59], improving aerobic fitness [15], and applying ventilation [49]. Extending the exposure time to thermal stress leads to an increase in core body temperature and dehydration levels [60]. Acclimatized workers, with beneficial physiological adaptations like an efficient sweating system, lower heart rate, and core body temperature, can tolerate higher levels of dehydration and lose more water through sweat per shift. This means that the maximum allowable exposure time is greater for acclimatized workers compared to non-acclimatized workers [38, 42, 47]. Venugopal et al. demonstrated a strong correlation between physical workload, thermal stress exposures, Heat Strain Indicators (HSIs), and HRIs, leading to adverse health outcomes among outdoor workers [46]. There is a pressing need for evidence-based reviews and interventions to prevent occupational heat stress and enhance comprehensive resilience labor policies for outdoor workers in low and middle-income countries as climate change progresses. Increased awareness and consciousness among workers can lead to better adaptability to climate change risks [31]. Workers often implement conscious and flexible behavioral attitudes to manage their heat stress, especially in extremely hot workplaces, such as outdoor work [49]. Understanding the relationship between endurance time and WBGT values is crucial for training workers in very hot environments and ensuring their health and safety [43]. Elevated carbon emissions in the atmosphere contribute to extremely hot environments and climate changes, exacerbating occupational heat strains for outdoor workers [61]. A high-quality air and work environment can enhance worker safety, health, productivity, and company profitability [37, 49]. Sustainable adaptation to warming climatic conditions [13] and social protection strategies during exposure to occupational heat stress depend on the availability of financial resources and collaborative efforts to overcome adaptation barriers [48]. The severity of occupational heat stress caused by climate change depends on workers’ sensitivity and vulnerability to different weather conditions. Additionally, the extent of adaptation capacity and resilience planning plays a crucial role [33, 38]. Also, establishing a program that can assess how thermal stress due to climate change may increase heat-related effects on outdoor workers and document future heat-related events leading to relevant occupational health and safety regulations, seems essential [15].

The HEAT-SHIELD project is a customized occupational heat stress-related warning system that provides short- and long-term heat warnings to safeguard workers’ health and productivity. This project represents a useful adaptation strategy aimed at protecting workers, particularly those exposed to the effects of climate change [55, 62–66].

The findings of this study are valuable for policymakers and professionals in the field of occupational health. They can use this information to develop guidelines and regulations aimed at preventing occupational heat stress and strengthening the resilience of outdoor workers during exposure to heat stress caused by climate change. However, it’s important to note that developing countries face a higher risk of negative occupational health outcomes compared to developed countries due to their lower adaptive capacity [46], increased poverty, and insufficient technological progress to combat climate change-induced temperature increases [6, 47]. Outdoor workers often lack awareness of heat-related risks and HRI due to global warming [67, 68]. Therefore, there is a critical need to raise awareness of heat-related hazards, bolster heat stress education, and update existing heat prevention measures. This includes optimizing current heat-related laws and adaptation policies to ensure effective implementation and compliance, especially in hot-dry and hot-humid work environments, particularly in low-middle-income countries [44, 48]. Studies of this nature are essential among workers in these countries to provide health professionals and senior managers with the necessary knowledge to inform occupational heat stress adaptation policies, social protection measures, and resilience strategies for sustainable development.

### Limitations

One limitation of this systematic review was the limited focus on female workers. Consequently, the results may not accurately represent the perspectives of women working outdoors, which is an important demographic to consider. Another significant limitation of this study is its heavy reliance on cross-sectional and experimental studies. Incorporating clinical aspects into data collection could greatly enhance and advance occupational health interventions. Furthermore, there is an evident scarcity of research exploring the social dimensions and the broader effects of occupational heat stress. Additionally, there is insufficient investigation into the adaptation strategies employed by workers in the context of increasing thermal stress and climate change, particularly in tropical and subtropical countries. These research gaps highlight the need for further studies to provide a more comprehensive understanding of this critical occupational health issue.

## Conclusion

Addressing the health risks associated with occupational thermal stress among outdoor workers requires a multi-level approach that includes standard procedures and safety interventions. Currently, there is a lack of formal guidelines for outdoor workers, and most advisory systems do not adequately support this workforce in implementing solutions to mitigate occupational heat stress and enhance climate change resilience. While many workers acknowledge the importance of increased hydration and clothing adjustments during hot-dry and hot-humid climate conditions, a smaller proportion attempt to modify the nature of their work or seek rest in cooler areas. It is crucial to recognize that occupational heat stress remains a prevalent issue among these populations. To address these challenges, we recommend conducting further research to enhance our understanding of strategies aimed at bolstering the resilience of outdoor workers against heat stress resulting from climate change. This research should encompass diverse fields such as medicine, climatology, occupational health, and epidemiology. Additionally, there is a need to improve information dissemination, develop relevant regulations, and implement protective strategies among outdoor workers. These efforts will aid in identifying and preventing heat stress-related policies, including mitigation and adaptation measures.

### Electronic supplementary material

Below is the link to the electronic supplementary material.

Supplementary Material 1

## Data Availability

The datasets used and analyzed during the current study are available from the corresponding author on request.
