# Supplementary material for: Climate change and heat stress resilient outdoor workers: findings from systematic literature review
Source: BMC Public Health. 2024 Jun 26;24:1711. doi: 10.1186/s12889-024-19212-3 (PMC11210127; doi:10.1186/s12889-024-19212-3)
Supplement: Supplementary file 1 — Supplementary Material 1 [file 12889_2024_19212_MOESM1_ESM.pdf]

Appendix١. Search strategy for Climate change and heat stress resilient workforce: findings from systematic literature review

SCOPUS

TITLE-ABS-KEY(“heat wave” OR “heat stress” OR “climate change*” OR (climate AND change) OR “extreme weather” OR “extreme heat” OR “global warming” OR “hot day*” OR “warm day*”) AND TITLE-ABS-KEY("heat tolerance" OR "heat resilien*" OR (heat AND resilien*) OR (heat AND tolera*) OR “Heat resistan*” OR thermotolerance OR “heat endurance” OR (heat AND endur*)) AND TITLE-ABS-KEY(worker* OR Firefighter* OR “fire fighter*” OR firem* OR “fire m*” OR nurs* OR operator* OR driver* OR farmer* OR welder* OR miner* OR employee OR laborer*)

Web of Science

TS=(“heat wave” OR “heat stress” OR “climate change*” OR (climate AND change) OR “extreme weather” OR “extreme heat” OR “global warming” OR “hot day*” OR “warm day*”) AND TS=("heat tolerance" OR "heat resilien*" OR (heat AND resilien*) OR (heat AND tolera*) OR “Heat resistan*” OR thermotolerance OR “heat endurance” OR (heat AND endur*)) AND TS=(worker* OR Firefighter* OR “fire fighter*” OR firem* OR “fire m*” OR nurs* OR operator* OR driver* OR farmer* OR welder* OR miner* OR employee OR laborer*)

PUBMED:

(“heat wave”[tiab] OR “heat stress”[tiab] OR “climate change*”[tiab] OR (climate[tiab] AND change[tiab]) OR “extreme weather”[tiab] OR “extreme heat”[tiab] OR “global warming”[tiab] OR “hot day*”[tiab] OR “warm day*”[tiab]) AND ("heat tolerance"[tiab] OR "heat resilien*"[tiab] OR (heat[tiab] AND resilien*[tiab]) OR (heat[tiab] AND tolera*[tiab]) OR “Heat resistan*”[tiab]

OR thermotolerance[tiab] OR “heat endurance”[tiab] OR (heat[tiab] AND endur*[tiab])) AND (worker*[tiab] OR Firefighter*[tiab] OR “fire fighter*”[tiab] OR firem*[tiab] OR “fire m*”[tiab] OR nurs*[tiab] OR operator*[tiab] OR driver*[tiab] OR farmer[tiab]* OR welder*[tiab] OR miner*[tiab] OR employee[tiab] OR laborer*[tiab])

Scholar google

heat stress AND thermal resilience

heat stress AND thermal endurance

heat stress" AND thermal resistance

heat wave" AND "thermal resilience

heat wave" AND "thermal endurance

heat wave" AND "thermal resistance

climate change AND thermal resilience

climate change AND thermal endurance

climate change AND thermal resistance

global warming AND thermal resilience

global warming AND thermal endurance

global warming AND thermal resistance
